# Hepatitis B virus genotypes/subgenotypes in voluntary blood donors in Makassar, South Sulawesi, Indonesia

**DOI:** 10.1186/1743-422X-6-128

**Published:** 2009-08-19

**Authors:** Andi Utama, Theresia I Octavia, Rama Dhenni, Upik A Miskad, Irawan Yusuf, Susan Tai

**Affiliations:** 1 Molecular Epidemiology Division, Mochtar Riady Institute for Nanotechnology, Lippo Karawaci, Tangerang, Banten 15810, Indonesia; 2 Faculty of Medicine, Hasanuddin University, Makassar, South Sulawesi 90245, Indonesia

## Abstract

**Background:**

Hepatitis B virus (HBV) genotype appears to show varying geographic distribution. Molecular epidemiological study of HBV in particular areas in Indonesia is still limited. This study was aimed to identify the prevalence of HBV genotype/subgenotype and mutations in basal core promoter (BCP) region in voluntary blood donors in Makassar, one of the biggest cities in east part of Indonesia.

A total of 214 hepatitis B surface antigen (HBsAg)-positive samples were enrolled in this study. HBV genotype/subgenotype was identified by genotype-specific PCR method or direct sequencing of pre-S region. Mutations in BCP were identified by direct sequencing of the corresponding region.

**Results:**

HBV/B and HBV/C were detected in 61.21% and 25.23% of the samples, while mix of HBV/B and HBV/C was found in 12.62% of the samples. Based on pre-S region, among HBV/B and HBV/C, HBV/B3 (95.00%) and HBV/C1 (58.82%) were predominant. Interestingly, HBV/D was identified in two samples (22.165.07 and 22.252.07). Complete genome sequences of two HBV/D strains (22.165.07 and 22.252.07) demonstrated that both strains belong to HBV/D6, and the divergence between the two strains were 1.45%, while divergences of both 22.165.07 and 22.252.07 strains with reference strain (AM422939/France) were 2.67%. A1762T/G1764A mutation was observed in 1.96% and 5.36%, whereas T1753V mutation was found in 2.94% and 1.79% of HBV/B and HBV/C, respectively.

**Conclusion:**

HBV/B and HBV/C are dominant in Makassar, similar to most areas in Indonesia. Mutations in BCP which might be associated with severity of liver disease are less common.

## Background

Hepatitis B virus (HBV) infection is a major health problem leading to significant morbidity and mortality worldwide. Approximately, two billion people in the world have been infected by HBV [1]. The majority of acute HBV infections are self-limiting, whereas chronic HBV infection can cause chronic hepatitis, liver cirrhosis, or hepatocellular carcinoma. It is well known that Indonesia has a moderate to high endemicity of hepatitis B virus (HBV) infection [2], due to the lack of proper health facilities or poor economical status and less public awareness.

HBV, a member of the *Hepadnaviridae*, is a relaxed circular double-stranded DNA virus, and is currently classified into eight genotypes (A-H) based on a comparison of the entire HBV genomic sequence [3]. HBV genotypes appear to show varying geographic distribution. For instance, HBV/A is prevalent in Europe, Africa, and India [4,5]. HBV/B and HBV/C are predominant in most part of Asia, including China and Japan [4,6]. The HBV/D is common in the Mediterranean area, the Middle East and India, whereas the HBV/E is localized in sub-Saharan Africa [4-10]. The HBV/F and HBV/H is only identified in Central and South America [4,11-13]. The HBV/G has been found in France, Germany and United States [14-16].

Molecular epidemiological studies of HBV, either a nationwide study or study on particular areas in Indonesia, have shown that HBV/B and HBV/C are the most prevalent genotypes in Indonesia [2,17-20], although HBV/A and HBV/D have also been found in Eastern Indonesia, such as Moluccas and Papua [19,20]. Particularly in South Moluccas, the prevalence of HBV/D is high, ranging from 50–88% [20]. In the same study, analysis of 12 samples from Makassar demonstrated that only genotype B and C were found in the samples [20]. To date, there is no comprehensive study about HBV genotype prevalence as well as the genetic analysis of HBV circulated in Makassar. In this study, we have analyzed HBV genotype/subgenotype and mutations in BCP region from voluntary blood donors in Makassar, which is located in Wallace territory and the biggest city in Eastern Indonesia.

## Methods

### Samples

Serum samples from 214 blood donors (age 18–64 years, mean 30.9 ± 10.3 years, male/female 153/61) which were positive for HBsAg were collected in the Blood Transfusion Unit, Red Cross Makassar, South Sulawesi, Indonesia, between February and August 2007. HBsAg was determined by commercially available kit (AxSYM^® ^HBsAg, Abbott Laboratories, Chicago, IL, USA). Blood samples were separated into sera and stored at -70°C until use. The study was approved by the Institutional Ethic Committee and informed consent was obtained from each donor.

### Viral DNA extraction and genotyping

HBV DNA was extracted from 200 μl serum using QIAamp^® ^DNA blood mini kit (Qiagen, Hilden, Germany) according to the manufacturer's instruction, and 50 μl eluted DNA was stored at -70°C until use. HBV genotyping was performed by PCR using genotype specific primers as described by Naito et al. [21]. To avoid false-positive results, instruction to prevent cross-contaminations were strictly followed, and results considered valid only when they were obtained in duplicate. Pre-S region was amplified by nested PCR using PCR Core System (Promega, Madison, WI, USA) and two sets of primers as previously described with minor modifications [22]. The first round PCR was performed for 35 cycles of 95°C for 1 min, 46.7°C for 30 s and 72°C for 1 min. The second round PCR was carried out similar to the first round PCR, except with an annealing temperature of 44.6°C. Primers PS1/PS2 and PS3/PS4 were used for the first and second rounds PCR, respectively. The PCR products were purified with Wizard^® ^SV Gel and PCR Clean-Up System (Promega, Madison, WI, USA), directly sequenced employing an ABI 3130xl Genetic Analyzer (Applied Biosystems, Inc., Foster City, CA, USA) with the Big Dye Terminator V3.1 Cycle Sequencing kit (Applied Biosystems, Inc.) using primers P3 and P4. Multiple alignments of pre-S sequences were done using the CLUSTAL W method [23]. Phylogenetic trees were constructed using Neighbor-Joining method [24] with Kimura's two-parameter [25] and 1,000 replicates of bootstrap resampling as implemented in MEGA 4.1 [26]. Subgenotypes were assigned as described previously [19,27,28].

### Full sequencing of HBV genotype D

The complete genome of HBV was amplified as 7 overlapping fragments (fragment 1–7) using nested-PCR with Go Taq PCR Core System (Promega). The list of primers and PCR products are shown in Table 1. First and second round PCR were performed for 35 cycles with same condition except for annealing temperature. The condition was as follow: denaturation at 95°C (5 min), annealing at 48.1–57.4°C (30 s) for the first round and 46.1–57.4°C (30 s) for the second round, and elongation at 72°C (1 min). PCR product was purified from agarose gel using Wizard^® ^SV Gel and PCR Clean-Up System (Promega), according to manufacturer's protocol, and directly sequenced. Percentage divergence of nucleotide sequences among HBV genotype D strain was calculated using MEGA 4.1 [26]. Phylogenetic trees were constructed similarly as in the pre-S sequences.

**Table 1:** Primers used for fragments amplification and sequencing of complete genome of HBV genotype D

**Fragment no. (bp)**	Primers*	**Polarity**	**Sequence (5'→'3)**	**Domain**	**Position**
1 (379)	HBPr1	Forward	GGGTCACCATATTCTTGGG	HBPol	2850–2860
	HBPr2	Forward	GAACAAGAGCTACAGCATGGG	HBPol/PreS1	2867–2888
	HBPr3	Reverse	CCACTGCATGGCCTGAGGATG	PreS1/PreS2/HBPol	3226–3246

2 (891)	HBPr14	Forward	TGGGGTGGAGCCCTCAG	PreS1/HBPol	3104–3120
	HBPr94	Reverse	GGTAWAAAGGGACTCAMGATG	HBPol/HBsAg	775–795
	HBPr135	Reverse	CARAGACAAAAGAAAATTGG	HBPol/HBsAg	803–822

3 (541)	HBPr440	Forward	TATGGATGATGTGGTATTGGG	HBPol/HBsAg	738–758
	HBPr113	Reverse	CCGGCAGATGAGAAGGCACAGACGG	HBX/HBPol	1549–1574
	HBPr374	Reverse	GTTCCGCAGTATGGATCGGCAGAGG	HBPol	1255–1279

4 (319)	HBPr110	Forward	CCTCTGCCGATCCATACTGCGGAAC	HBPol	1255–1279
	HBPr113	Reverse	CCGGCAGATGAGAAGGCACAGACGG	HBX/HBPol	1549–1574

5 (560)	HB1	Forward	GCCAAGTGTTTGCTGACGC	HBPol	1174–1192
	HB2	Forward	CCATACTGCGGAACTCCTAG	HBPol	1265–1284
	HB3	Reverse	AAAGTTGCATGGTGCTGGTG	HBX	1803–1822

6 (726)	HBPr86	Forward	ACATAAGAGGACTCTTGGAC	HBX	1652–1671
	HBPr87	Forward	TACTTCAAAGACTGTGTGTTTA	HBX	1704–1723
	HBPr111	Reverse	CTGCGAGGCGAGGGAGTTCTTCTTC	Core/HBPol	2406–2430

7 (585)	HBPr33	Reverse	CTGAGGGCTCCACCCCA	PreS1/HBPol	3104–3120
	HBPr446	Forward	GGAGTGTGGATTCGCACTCC	Core	2303–2323
	HBPr448	Reverse	CCCATGCTGTAGCTCTTGTTC	HBPol/PreS1	2868–2888

* All primers, except HB1–HB3, were same as previous report [15].

### BCP Mutations analysis

BCP region was amplified by hemi-nested PCR using HBPr86/HBPr7 and HBPr87/HBPr 7 for two rounds PCR as previously described, with a slightly modified touch-down PCR [15]. The following cycling parameters were used for PCR: denaturation at 95°C (30 s), annealing at 60.5–53.5°C (30 s) and elongation at 72°C (30 s). For the first 15 cycles, the annealing temperature was 60.5°C; this temperature was then reduced by 0.5°C per cycle, and continued with constant annealing temperature for another 30 cycles. The first and second rounds PCR were performed similarly except for annealing temperature (58.5–51.5°C). The PCR products were purified with Wizard^® ^SV Gel and PCR Clean-Up System (Promega) and directly sequenced using primer HBPr7. Multiple alignments of BCP sequences were done using the same method as in the pre-S sequences.

## Results

### HBV genotype prevalence

**Figure 1:**
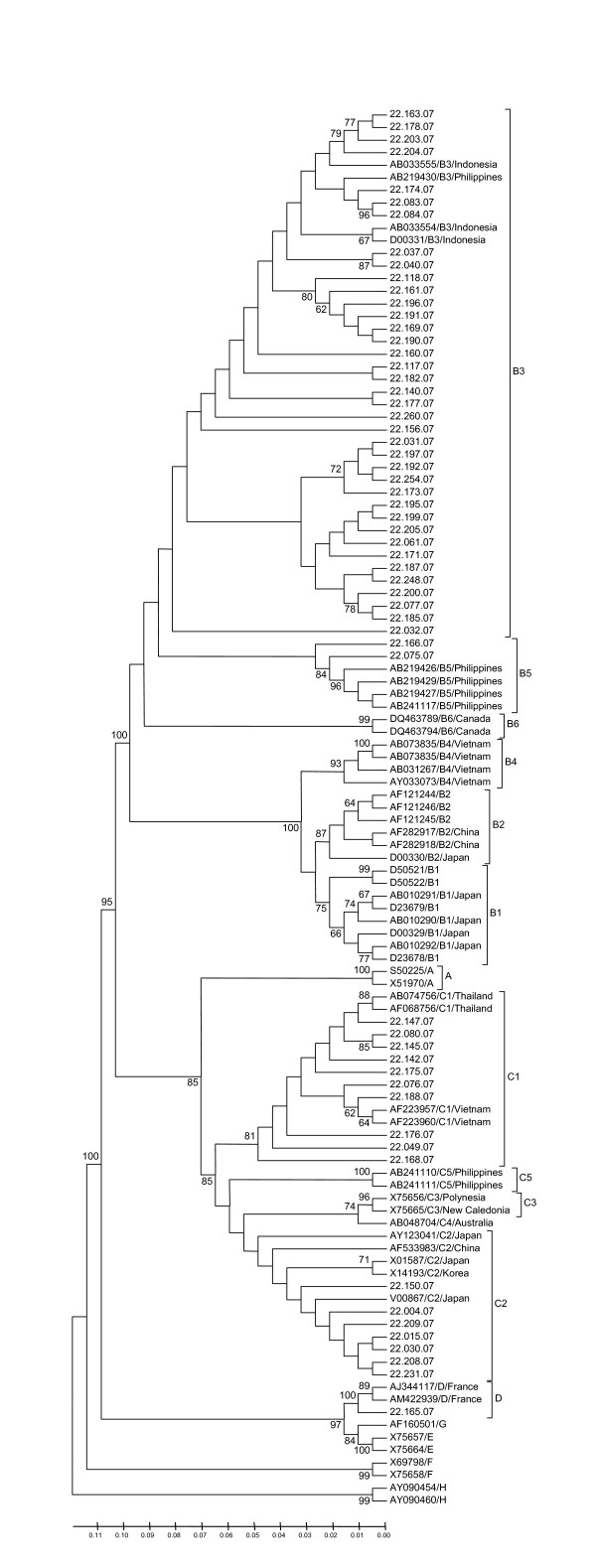
**Phylogenetic analysis of pre-S region of HBV**. Phylogenetic tree was constructed from the pre-S sequences of 58 samples (accession number EU926161–EU926217) together with sequences retrieved from GenBank.

Of the 214 subjects enrolled in this study, 156 samples (72.90%) could be genotyped by genotype-specific PCR, whereas 58 samples (27.10%) could not. Based on this method, it was found that 91 samples (58.33%) were HBV/B, 37 samples (23.72%) were HBV/C, 27 samples (17.31%) were mix of HBV/B and HBV/C, and one sample (0.64%) was HBV/D (Table 2). For 58 samples that could not be identified by genotype-specific PCR, the pre-S region was amplified and directly sequenced. Of 58 samples, 40 samples (68.97%) were HBV/B, and 38 of those HBV/B (95.00% of HBV/B or 65.52% of all samples) were HBV/B3 and the other two isolates were HBV/B5 (Fig. 1, Table 2). Seventeen (29.31%) of 58 samples were HBV/C, which was divided into HBV/C1 (58.82% of HBV/C or 17.24% of all samples) and HBV/C2 (41.18% of HBV/C or 12.07% of all samples). In addition, HBV/D was only found in one sample. From HBV genotyping based on both genotype-specific PCR and pre-S sequence on 214 blood donors, it was shown that HBV/B was predominant (131 samples, 61.21%), followed by HBV/C (54 samples, 25.23%), mix of HBV/B and HBV/C (27 samples, 12.62%), and HBV/D (2 samples, 0.93%) (Table 2).

**Table 2:** HBV genotype analysis of samples based on genotype specific PCR and pre-S sequence.

	**No. (%) based on**		
	
**Genotype/Subgenotype**	**Genotype-specific PCR**	**Pre-S sequence**	**All**
B	91 (58.33)	40 (68.97)	131 (61.21)
B3		38 (65.52)	
B5		2 (3.45)	
C	37 (23.72)	17 (29.31)	54 (25.23)
C1		10 (17.24)	
C2		7 (12.07)	
B + C	27 (17.31)	0 (0.00)	27 (12.62)
D	1 (0.64)	1 (1.72)	2 (0.93)

Total	156 (100.00)	58 (100.00)	214 (100.00)

### Full sequence of HBV genotype D

**Figure 2:**
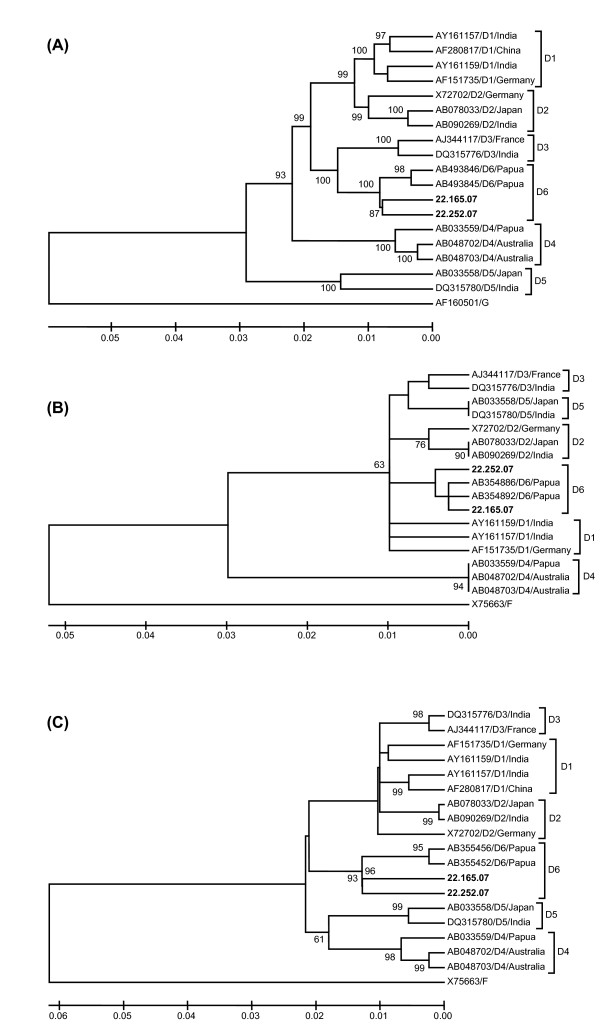
**Phylogenetic analysis of complete genome (A), partial S (B) and pre-C/C (C) of HBV/D isolate 22.165.07 and 22.252.07 (accession number **EU921418 and EU921419**)**. Phylogenetic trees were constructed from sequences of each region of HBV genome from the samples and sequences retrieved from GenBank. The sequences used for partial S and pre-C/C were nucleotide no. 500–703 and 1814–2452, respectively, as described in previous report [19].

Since HBV/D is rare in this area, we interested to analyze the complete genome of the two HBV/D strains (22.165.07 and 22.252.07) found in the samples. Phylogenetic analysis of complete genome, partial S, and pre-C/C regions confirmed that the two viruses were HBV/D6 (Fig. 2). Percentage divergences of complete genome, pre-S/S, X, pre-C/C, and Pol genes among the two strains were 1.45%, 1.45%, 1.40%, 0.43%, and 2.19%, respectively. Based on the complete genome, divergences of 22.165.07 strain with reference strains, AM422939 (France), AB493846 (Papua), and AB493845 (Papua) were 2.67%, 1.44% and 2.03%, respectively (Table 3). Similarly, another strain (22.252.07) showed 2.67%, 1.18%, and 1.90% divergences with those three reference strains. Based on each gene, the two present strains demonstrated high identity with AB493846 (Papua) in X genes, with identity of 99.57–100.00%, but less homology with AM422939 (France) in pre-C/C region, which showed only 95.62% identity (Table 3).

**Table 3:** Percentage divergences of nucleotide sequences between present and reference strains

	**22.165.07**				**22.252.07**			
	
**Region**	**22.252.07**	AM422939** (France)**	AB493846** (Papua)**	AB493845** (Papua)**	**22.165.07**	AM422939** (France)**	AB493846** (Papua)**	AB493845** (Papua)**
Complete Genome	1.45	2.67	1.44	2.03	1.45	2.67	1.18	1.90
Pre-S/S	1.45	1.79	2.55	2.55	1.45	1.88	2.39	2.71
Polymerase	1.40	2.16	1.25	1.65	1.40	2.20	1.02	1.57
X gene	0.43	1.72	0.43	2.45	0.43	1.29	0.00	2.00
Pre-C/C	2.19	4.38	1.23	2.13	2.19	4.38	0.97	1.95

### Genetic variation in BCP region

**Figure 3:**
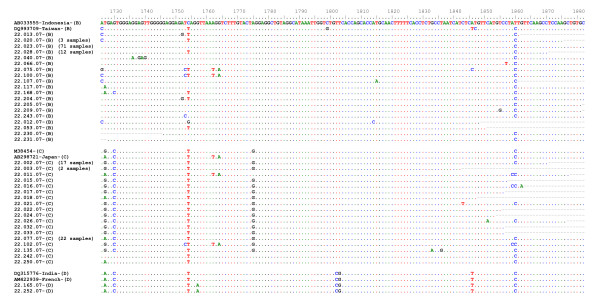
**Alignment of BCP sequences**. The BCP sequences of HBV/B of 102 samples (accession number EU938143–EU938244), HBV/C of 56 samples (accession number EU938245–EU938300), and HBV/D of 2 samples (accession number EU921418 and EU921419) were aligned.

In order to find out specific nucleotide substitution among HBV in the subjects, BCP region was analyzed. Of 214 samples, 169 were positive for PCR and sequencing, however 9 samples could not be analyzed due to incomplete data. BCP sequence of HBV/B (102 samples), HBV/C (56 samples), and HBV/D (2 samples) were respectively aligned. It was observed that overall nucleotide substitution was rarely occurred in all genotypes. Double mutation (A1762T/G1764A), one of significant mutations associated with advanced liver disease including HCC, was only found in 1.96% (2/102) of HBV/B (Fig. 3). Likewise, the double mutation was only observed in 5.36% (3/56) of HBV/C (Fig. 3). Analysis of nucleotide at position 1753 showed that a T-to-V (A/G/C) mutation, which also suggested having association with liver disease progression, was found as much as 2.94% (3/102) and 1.79% (1/56) in HBV/B and HBV/C, respectively. None of these mutations was identified in HBV/D (Fig. 3). However, a G-to-A substitution at nucleotide 1896, which prevents the production of HBeAg by introducing a premature stop codon into the open reading frame of the pre-C region, was found in both strains of HBV/D (data not shown).

## Discussion

The present study demonstrates that HBV/B (61.21%) and HBV/C (25.23%) were the most prevalent among HBsAg-positive blood donors in Makassar, South Sulawesi, although HBV/D was also rarely found in the samples. Analysis of pre-S sequence of 58 samples revealed that in HBV/B the percentage of HBV/B3 was much higher than HBV/B5. In HBV/C, on the other hand, HBV/C1 and HBV/C2 were detected with similar frequency.

HBV/B3 is widely distributed in Indonesia, but has not been reported in other countries, suggesting that HBV/B3 is indigenous to Indonesia [20]. The HBV types we found in Makassar are similar to those reported in previous studies from several areas in Indonesia [2,17-19], but the frequencies are different to Papua and Moluccas in where HBV/C is more dominant [19,20]. In addition, no HBV/A was found in Makassar, although it was found in Balikpapan and Kupang [20].

Several studies have reported the prevalence of HBV genotype from blood donors in the southern Asian region. A study from Thailand demonstrated that HBV/A, HBV/B and HBV/C were detected among blood donors, where HBV/C (89.3%) was the most prevalent compared to HBV/B (7.4%) and HBV/A (0.5%) [29]. Although this study did not identify the subgenotype, complete genome analysis of HBV/C from Thailand and some other countries which are geographically close to Indonesia such as Vietnam and Myanmar showed that most of HBV/C was classified into HBV/C1 [28], whereas in Makassar HBV/C1 and HBV/C2 were dominant. On the other hand, analysis of asymptomatic HBV carriers from the Philippines showed that the prevalence of HBV/B, HBV/C, HBV/D, mix of HBV/B and HBV/D, and HBV/A were 53.4, 21.4, 14.3, 7.1, and 3.6%, respectively [30]. In general, the percentage of HBV/B in the Philippines was similar to that in Makassar, although it is not possible to compare the subgenotypes with the available data. However, all HBV/C strains in the Philippines samples were classified into HBV/C5, which is different with subgenotypes circulated in Makassar (HBV/C1 and HBV/C2). A study from Malaysia using small number of healthy blood donors and chronic hepatitis B carriers demonstrated that HBV/C, HBV/B, and HBV/D were identified in the samples [31], which is quite similar to our findings in Makassar.

Studies from India demonstrated that the distribution of HBV genotype in blood donors was slightly different between Eastern and Southern India. In Eastern India, beside the major HBV genotypes transmitted in India such as HBV/D (56.0%) and HBV/A (20.6%), quite high percentage of HBV/C (23.4%) was also found in the blood donors [32]. Phylogenetic analysis revealed that those HBV/C strains that clustered with HBV/C found in South-East Asia including Indonesia were classified as HBV/C1 [32]. On the other hand, in Southern India, only HBV/D (76.11%) and HBV/A (11.94%) were detected [33], similar to the HBV genotype distribution pattern in Northern and Western India [5].

In China, HBV/C was detected in high percentage (68.0%) from blood donors along with HBV/B (25.8%), HBV/A (2.1%) and mix HBV/B and HBV/C (4.1%), although the subgenotype was not identified in this study [34]. Thus, in general, the distribution of HBV genotype from blood donors in Makassar is different to that in India and China.

Prior to our study, in Indonesia HBV/D has only previously been found in Papua and Moluccas [19,20]. Therefore, we were interested to analyze the complete genome of the two HBV/D strains (22.165.07 and 22.252.07) found in our samples from Makassar. Phylogenetic analysis of complete genome, partial S, and pre-C/C regions confirmed that the two HBV/D strains were subgenotype D6 (Fig. 2). The present HBV/D strains were then compared with two references recently published from Papua (AB493846 and AB493846) and one reference which found in France (AM422939). Based on the complete genome, divergences between the present strains and Papua strains (HBV/D6) were lower than the divergence between the two strains and France strain (HBV/D3) (Fig. 2). Thus, it is confirmed that the HBV/D found in Makassar was the same with the HBV/D strains present in Papua (HBV/D6) [19]. Because a previous study found HBV/D strains in Moluccas were HBV/D1 and HBV/D3 [20], it is possible that the HBV/D found in Makassar has been imported from Papua, not Moluccas, even though Moluccas is very close to Makassar.

Many studies have demonstrated that HBV mutations, including mutations in BCP region, are linked with the severity and outcome of HBV infection [35-37]. Our recent data also revealed that mutations in BCP were associated with clinical outcome of liver disease [38]. We therefore looked for mutations in the BCP region. Double mutation (A1762T/G1764A) was only found in 1.96% of HBV/B (Fig. 3). Likewise, the double mutation was only observed in 5.36% of HBV/C (Fig. 3). Analysis of the nucleotide at position 1753 showed that a T-to-V (A/G/C) mutation, which has also been associated with HCC development [39], was found to be less frequent in both HBV/B and HBV/C (2.94% and 1.79, respectively). None of these mutations was identified in HBV/D (Fig. 3). Hence, our results demonstrated that the frequency of mutations in BCP in blood donors in Makassar was low. In our previous study, we included 15 HBV-associated liver disease samples from Makassar [38], and found that the prevalence of T1753V and A1762T/G1764A mutations were 40.0% and 60.0%, respectively, suggesting that those mutations were associated with severity of liver disease. Since both T1753V and A1762T/G1764A mutations were less common in blood donors, the chance of developing severe liver disease might be relatively low in the blood donors in Makassar.

In comparison, a study from Indian blood donors showed that the occurrence of double mutation in BCP was extremely high in HBV/C (72.4%) and relatively high in HBV/A (24.1%) and HBV/D (21.5%) [32]. Another study also reported that K130M and V131I substitutions, which are corresponding to double mutation in BCP, from Indian inactive carrier were generally high (36.0%) [40]. However, they found that the occurrence of other mutations suggested to have associated with HCC was low [40]. If BCP mutation is strongly associated with clinical outcome of liver disease including HCC, the incidence of HCC must be high in India. In fact, the HCC incidence was very low in India [41], thus the association of BCP mutations as well as other mutations with severity of liver disease might be not depend only on virus mutations, but also host factors, and therefore needs further investigation.

## Conclusion

HBV/B and HBV/C are dominant in Makassar, similar with the HBV genotype distribution in most areas in Indonesia. Mutations in BCP which might be associated with severity of liver disease are less common.

## Competing interests

The authors declare that they have no competing interests.

## Authors' contributions

Conceived of the study, participated in its design and coordination, drafted the manuscript and coordinate the whole work team: AU. Carried out the molecular genotyping study: TIO. Responsible for sample and clinical data collection, and contributed in data analysis: RD. Participated in sample and clinical data collection: UAM. Participated in the editing the manuscript and clinical data: IY. Coordinated the research effort and participated in manuscript preparation: ST. All authors have read and approved the final manuscript.
